# Structural and Pharmacological Characterization of AT-121 Reveals Carbonic Anhydrase Inhibition as a Complementary Mechanism to Dual MOR/NOR Agonism

**DOI:** 10.1021/acsmedchemlett.6c00147

**Published:** 2026-04-19

**Authors:** Alessandro Bonardi, Marta Ferraroni, Paola Gratteri, Claudiu T. Supuran, Andrea Angeli

**Affiliations:** † NEUROFARBA Department, Sezione di Scienze Farmaceutiche, 9300 University of Florence, Via Ugo Schiff 6, 50019 Sesto Fiorentino, Florence, Italy; ‡ Department of Chemistry “Ugo Schiff”, University of Florence, Via della Lastruccia 3-13, 50019 Sesto Fiorentino, Florence, Italy

**Keywords:** Carbonic Anhydrase, Metalloenzyme, AT-121, μ-opioid receptor, nociceptin receptor, sulfamide

## Abstract

AT-121 is a bifunctional ligand acting as a partial agonist at both μ-opioid and nociceptin receptors, designed to retain potent analgesia while reducing opioid-associated side effects. Carbonic anhydrases (CAs) catalyze the reversible conversion of carbon dioxide to bicarbonate and protons and, in the central nervous system, play key roles in neuronal excitability, synaptic transmission, and pain processing. Inhibition profiling across all human CA isoforms demonstrated activity against several cytosolic enzymes, as well as membrane-associated isoforms hCA IV and XII, the latter showing the highest inhibitory potency (K_I_ = 70.1 nM). X-ray crystallography of the hCA II-AT-121 complex highlighting dual binding conformations mediated by an aliphatic sulfamide moiety within the enzyme active site. Complementary *in silico* docking simulations across additional isoforms supported these observations. Collectively, these findings suggest that AT-121 operates a multimodal mechanism combining dual opioid receptor activation with CA inhibition, supporting its potential as a safer, nonaddictive analgesic strategy.

## Graphical abstract

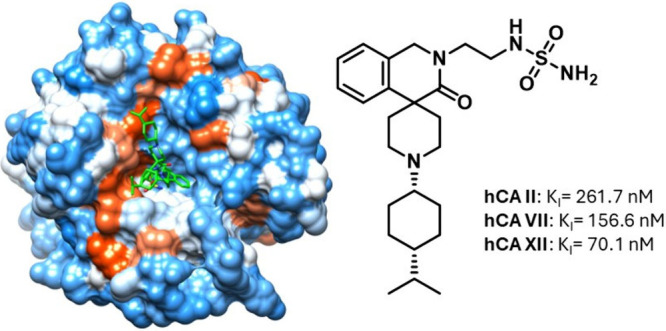

The dramatic increase in prescription opioid misuse, along with the growing incidence of opioid-related overdose fatalities, has imposed significant social and health burdens.^1,2^ Although opioids remain the most effective pharmacological option for treating severe pain, their long-term use is fundamentally limited by safety concerns.^3,4^ These include relatively common gastrointestinal disturbances, such as constipation, nausea and vomiting, as well as more serious and potentially lethal consequences, including respiratory depression, tolerance, addiction, increased risk of abuse, and overdose mortality.^5,6^ Consequently, the development of new therapeutic strategies that preserve opioid analgesia while mitigating these deleterious effects is a key priority in addressing this public health emergency.^7^ Several pharmacological strategies have been explored to address the dual challenge of effectively managing pain and opioid addiction. Although these efforts, the lack of analgesics that provide efficacy equal to that of opioids without the associated side effects remains one of the major contributing factors to the current opioid crisis. Until now, approved opioid drugs exert their therapeutic and side effects primarily through activation of the μ-opioid receptor (MOR).^8^ Consequently, despite decades of research advances in MOR pharmacology, the absence of safer alternatives has perpetuated dependence on drugs with narrow therapeutic windows.^9^ Significant efforts have been made to develop selective opioid receptor ligands that preferentially activate G protein-mediated signaling while minimizing β-arrestin recruitment.^10,11^ This approach emerged from evidence implicating β-arrestin pathways in several deleterious effects of MOR activation, including respiratory depression, constipation, and tolerance. Oliceridine (Figure 1), the latest opioid drug approved by the FDA in 2020, is an example of this strategy, demonstrating potent analgesia with an improved acute safety profile compared to morphine.^12,13^

**Figure 1:**
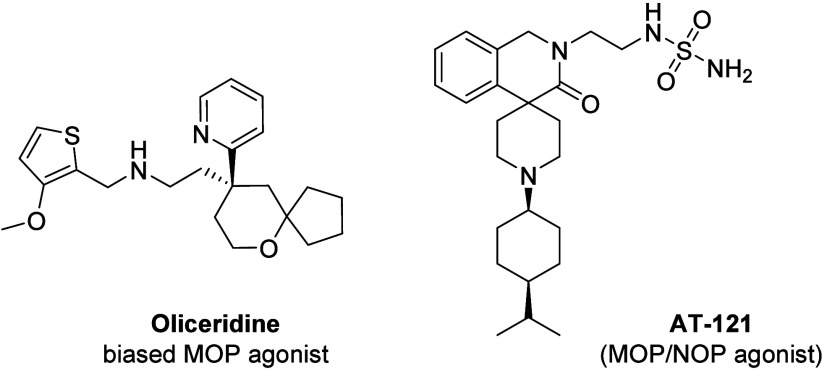
Chemical structures of oliceridine and AT-121.

However, subsequent preclinical and clinical findings revealed important limitations of this paradigm, including the persistence of abuse risk and incomplete separation of analgesia from respiratory and reinforcing effects upon repeated administration.^14,15^ As a result, attention has increasingly shifted toward dual receptor targeting, which can more effectively modulate complex neurobiological networks underlying pain, addiction, and opioid-related side effects. Beyond MOR, the activation of the nociceptin receptor (NOR) produces strong antinociception without many of the side effects typical of opioids.^16^ Bifunctional MOR/NOR agonists have emerged as a novel strategy for improving analgesic efficacy while attenuating the adverse effects associated with conventional MOR agonists.^17,18^ This concept challenges previous paradigms focused exclusively on receptor selectivity or partial signaling at a single receptor and instead supports rational polypharmacology as a means of improving therapeutic outcomes. One of the most promising examples of this approach is AT-121 (Figure 1), a nonmorphine bifunctional ligand with partial agonist activity on MOR/NOR, which produces potent antinociceptive and antiallodynic effects comparable to or superior to those of morphine, while exhibiting significantly reduced adverse effects, highlighting its potential usefulness in both pain management and opioid use disorder.^19,20^ The presence of a sulfamide moiety in the AT-121 scaffold, a bioisoster of sulfonamide group, raises our interest to investigate it as Carbonic Anhydrase (CA, EC 4.2.1.1) Inhibitor (CAI). Indeed, this metalloenzyme catalyzes the reversible conversion of carbon dioxide to bicarbonate and a proton^21^ and, in the central nervous system (CNS), this reaction is implicated in modulating neuronal excitability, synaptic transmission, and pain processing.^22,23^ Indeed, it has been shown that inhibition of specific CA isoforms affects ion channel function and modulates nociceptive signaling in a neuropathic pain model, as well as in opioid withdrawal, reducing drug-seeking behavior.^24–27^ Consequently, emerging evidence suggests that CA inhibitors may be investigated as potential adjuncts to opioid drugs to reduce side effects by indirectly influencing opioid receptor signaling pathways,^28^ providing a mechanistic rationale for combining CA modulation with opioid analgesics to achieve safer and more effective pain control.^29^ The inhibitory activity of AT-121 was evaluated against all catalytically active human (h) CA isoforms (hCA I–XIV) and compared with the reference inhibitor acetazolamide (AAZ) using a stopped-flow CO_2_ hydration assay (Table 1).

**Table 1:** Inhibition Data of All Catalytically Active Human CAs (hCA I–XIV) with AT-121 and AAZ by a Stopped-Flow CO_2_ Hydrase Assay

	K_I_ (nM)^a^	
compd	AT-121	AAZ
hCA I	4312	250.0
hCA II	261.7	12.1
hCA III	>10000	>10000
hCA IV	7498	74.0
hCA VA	>10000	63.0
hCA VB	>10000	54.0
hCA VI	1880	11.0
hCA VII	156.6	2.5
hCA IX	>10000	25.7
hCA XII	70.1	5.7
hCA XIII	>10000	17.0
hCA XIV	>10000	41.0

a Mean from 3 different assays, by a stopped flow technique (errors were in the range of ±5–10% of the reported values).

Among the cytosolic CA isoforms, AT-121 exhibited weak inhibitory potency against hCA I with a K_I_ value in the micromolar range (K_I_= 4312 nM). On the other hand, inhibition of the ubiquitously expressed hCA II was approximately 1 order of magnitude stronger with K_I_ of 261.7 nM. Notably, hCA VII was the most potently inhibited cytosolic isoform (K_I_= 156.6 nM) which is particularly relevant given its high expression in the CNS and its established role in neuronal excitability and pain processing. In contrast, no significant inhibition was observed for hCA III and hCA XIII (K_I_ > 10 μM) and AT-121 showed no measurable inhibition also for the mitochondrial isoforms hCA VA and hCA VB. The secreted isoform hCA VI was weakly inhibited by AT-121 (K_I_= 1880 nM). Among the membrane-bound isoforms, AT-121 demonstrated its highest inhibition potency against hCA XII (K_I_= 70.1 nM). In contrast, inhibition of hCA IV was markedly weaker (K_I_ = 7498 nM) and no inhibition was observed for hCA IX and hCA XIV. AAZ inhibited all tested isoforms with higher potency reflecting its well-established role as a pan-CA inhibitor. In contrast, AT-121 exhibited a narrower inhibition spectrum with reduced potency toward CA isoforms such as hCA I and hCA IV, while retaining good activity against CNS-relevant and pain-associated isoforms such as hCA II, VII and hCA XII.

Next, given the high expression of the cytosolic isoform hCA II in the CNS, we determined the X-ray crystal structure of hCA II in complex with AT-121 to elucidate its inhibitory interaction with this enzyme. Inspection of the active site revealed an electron density map fully consistent with the presence of AT-121 (Figure S1B). Notably, the inhibitor adopts two distinct conformations within the active site, sharing an identical zinc-binding mode but differing in the orientation of the scaffold tail, which is positioned in opposite directions relative to the hydrophobic pocket of hCA II (Figure S1A). This conformational duality appears to arise from the presence of an aliphatic sulfamide pharmacophore; in contrast, aromatic sulfamides have typically been reported to bind in a single conformation within the CA active site.^30,31^ As observed for other sulfamide-containing inhibitors, such as famotidine^30^ and the more recently reported aprocitentan,^31^ the deprotonated nitrogen atom of the sulfamide group displaces the zinc-bound water molecule, resulting in a tetrahedral coordination geometry on the catalytic zinc ion. Consistent with its sulfonamide bioisostere, the sulfamide moiety of AT-121 forms a conserved hydrogen bond between one of its oxygen atoms and the backbone amide of Thr199, contributing to complex stabilization.^32,33^ In the first binding conformation (Figure 2A), further stabilization is provided by a water-mediated hydrogen bond between the second nitrogen atom of the sulfamide group and Thr200; this residue also participates in a direct hydrogen bond with the carbonylic group of AT-121. The aliphatic chain engages in hydrophobic interaction with the side chain of Leu198. In this conformation, the aromatic ring participates in multiple hydrophobic contacts with His64, Ala65, and Thr200, as well as a π–π stacking interaction with His94. Additionally, the carbonylic group forms a water-bridged interaction with Pro201. Finally, the isopropylcyclohexane scaffold establishes hydrophobic contacts with the side chains of Phe131 and Pro202, further anchoring the ligand with the hydrophobic pocket of the active site.

**Figure 2:**
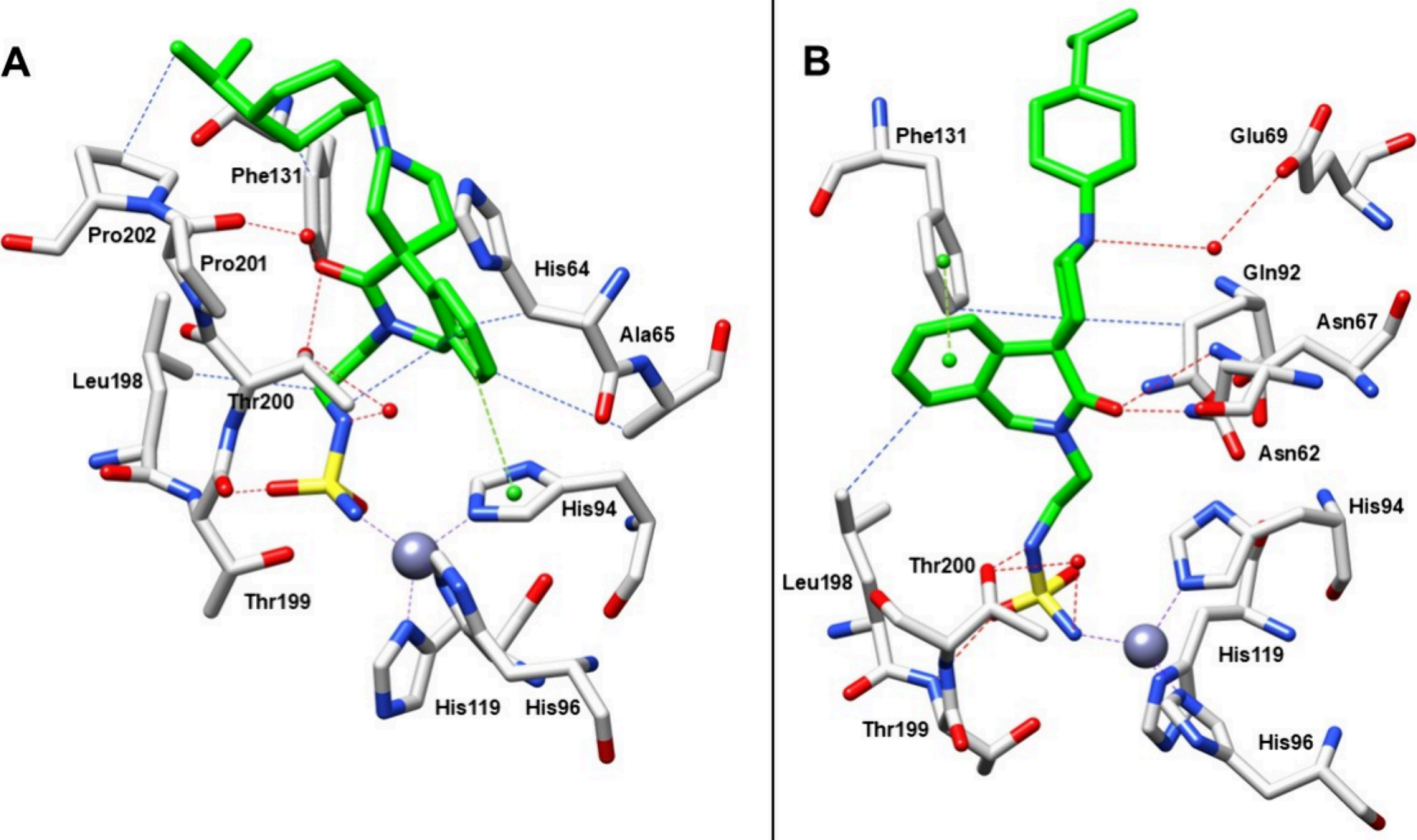
X-ray crystal structure of hCA II bound with AT-121. For clarity the two conformations of AT-121 were represented in two different images (A and B) (PDB: 29IR). Residues involved in the binding of inhibitor are also shown; the gray sphere represents the zinc ion in the active site of the proteins. The purple dotted lines show the coordination of the zinc ion, van der Waals interactions are shown in blue, hydrogen bonds and water bridge are shown in red, π-stacking interactions are shown in green.

The second binding conformation (Figure 2B) displays several notable differences compared with the conformation described above. In this arrangement, Thr200 forms a hydrogen bond with the nitrogen atom of the sulfamide group, while a water bridge interaction involving the deprotonated nitrogen further stabilizes the ligand within the active site. A distinct orientation of the aliphatic chain lost the hydrophobic interaction with Leu198, resulting in a reorientation of the dihydroisoquinolinone scaffold. This altered positioning enables the carbonylic group to form multiple hydrogen bonds with Asn62 and Asn67, while the aromatic ring engages in a π–π stacking interaction with Phe131. Additionally, the piperidine ring participates in hydrophobic interactions with the side chains of Gln92 and Phe131. In contrast to the first conformation, the isopropylcyclohexane scaffold does not contribute significantly to stabilizing interactions in this binding mode.

To obtain molecular-level information on the selectivity characteristics of AT-121, *in silico* docking simulations were performed across the remaining hCA isoforms. In particular, no inhibitor molecule were found to bind within the active sites of hCA III, VA, VB, IX, XIII, and XIV, consistent with the weak inhibitory activity observed experimentally against these isoforms. Instead, for hCA I, IV, VI, VII, and XII, all docking solutions showed the sulfamide moiety deeply bound within the catalytic site. In detail, the deprotonated primary nitrogen atom (RNHSO_2_NH^–^) coordinates the catalytic zinc ion, completing its tetrahedral geometry. This binding mode is consistent with literature reports and with the crystallographic structure of the AT-121–hCA II complex (Figure 2). The zinc coordination is further stabilized by two H-bonds involving the sulfamide S = O and NH groups, which interact with the backbone NH and the side chain OH of Thr199 (Thr220 in hCA VI), respectively. The dihydroisoquinoline aromatic ring contributes to binding through multiple van der Waals interactions and π–π stacking with His64 (hCA IV), His111 (hCA VI), and Phe131 (hCA VII). In hCA I, the weaker inhibitory profile can be attributed to steric constraints imposed by a narrower active site (Figure 3A). In contrast, in hCA IV and VI, suboptimal ligand–target complementarity arises from the presence of several charged (hCA IV) or polar residues (hCA IV and VI) in the middle and outer regions of the active site. This environment induces the 4-isopropylcyclohexyl moiety to fold inward, toward the catalytic cavity, where it forms van der Waals contacts with Leu198 (Figure 3B,C). Notably, in hCA VI, the carbonyl group of the dihydroisoquinoline core establishes two additional hydrogen bonds with Tyr149 and Gln109, accounting for the improved inhibitory activity relative to hCA II and IV (Figure 3C). In hCA VII, whose active site more closely resembles that of hCA II in both size and residue composition, π–π stacking with Phe131 directs the 4-isopropylcyclohexyl group toward Trp5, enabling extensive lipophilic interactions (Figure 3D).

**Figure 3:**
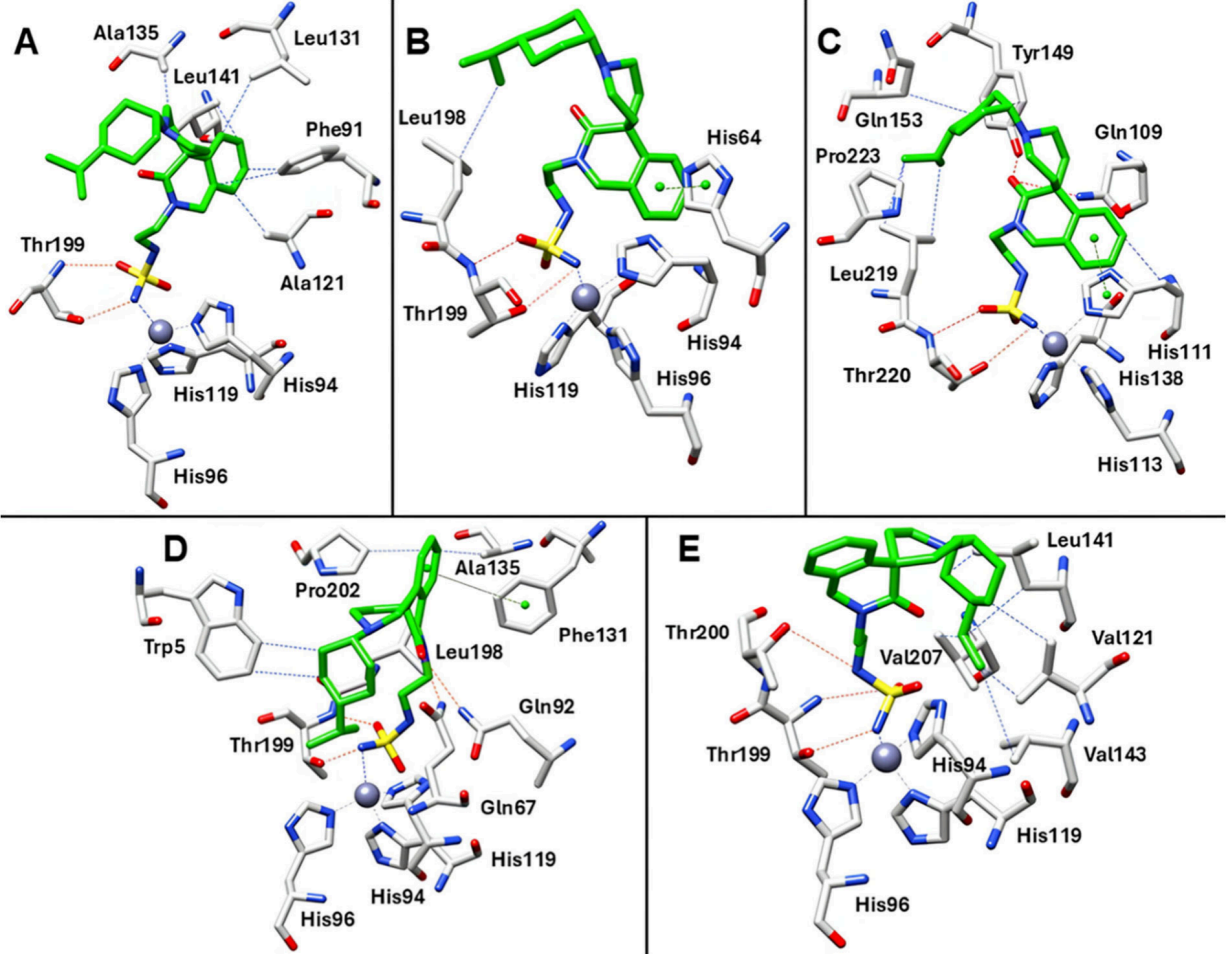
Binding mode of AT-121 (green) within (A) hCA I, (B) hCA IV, (C) hCA VI, (D) hCA VII, and (E) hCA XII. H-bonds, π-π stacking, and vdW interactions are depicted as red, green, and blue dashed lines. The zinc ion is represented as a gray sphere.

Additionally, the dihydroisoquinoline carbonyl group is positioned within hydrogen-bonding distance of the amide side chains of Gln67 and Gln92. In the larger active site of hCA XII, where steric hindrance is reduced, AT-121 forms an additional hydrogen bond between the secondary sulfamide NH and the side chain OH of Thr200, further stabilizing zinc coordination (Figure 3E). The 4-isopropylcyclohexyl substituent is oriented toward lipophilic residues (Val121, Leu141, and Leu207), maximizing hydrophobic contacts. To further refine the docking results, MM-GBSA calculations were performed to estimate the binding free energy (ΔG, kcal/mol), incorporating solvent effects and allowing refinement of residues within 3 Å of the ligand. The predicted binding energies followed the same trend observed experimentally in kinetic assays, yielding ΔG values of −29.64 kcal/mol (hCA I), −13.67 kcal/mol (hCA IV), −20.45 kcal/mol (hCA VI), −36.96 kcal/mol (hCA VII), and −48.69 kcal/mol (hCA XII), thereby supporting the computational model.

In summary, the pattern of CA inhibition, suggests that AT-121 targets several CA isoforms directly involved in neuronal function. However, hCA IV also if weak inhibited recently was demonstrated implicated in opioid withdrawal and in reducing drug-seeking behavior. When considered alongside its bifunctional MOR/NOR agonist activity, these findings support a multimodal mechanism of action in which CA inhibition may synergize with opioid receptor modulation to enhance analgesic efficacy while minimizing adverse effects.

## Supplementary Material



## Notes

Experimental sections, supplementary figures and X-ray data collection (PDF)

## Abbreviations

CA: carbonic anhydrase
MOR: μ-opioid receptor
NOR: nociceptin receptor
CAI: carbonic anhydrase inhibitor
AAZ: acetazolamide
CNS: central nervous system
