# Supplementary material for: Structural and Pharmacological Characterization of AT-121 Reveals Carbonic Anhydrase Inhibition as a Complementary Mechanism to Dual MOR/NOR Agonism
Source: ACS Med Chem Lett. 2026 Apr 19;17(5):1202–7. doi: 10.1021/acsmedchemlett.6c00147 (PMC13181446; doi:10.1021/acsmedchemlett.6c00147)
Supplement: Supplementary file 1 [file ml6c00147_si_001.pdf]

Supplementary Material for

Structural and Pharmacological Characterization of AT-121 Reveals Carbonic Anhydrase Inhibition as a Complementary Mechanism to Dual MOP/NOP Agonism

Alessandro Bonardi[a], Marta Ferraroni[b], Paola Gratteri[a], Claudiu T. Supuran[a], Andrea Angeli[a]*

[a] NEUROFARBA Department, Sezione di Scienze Farmaceutiche, University of Florence, Via Ugo Schiff 6, 50019, Sesto Fiorentino, Florence, Italy.

[b] Department of Chemistry "Ugo Schiff", University of Florence, Via della Lastruccia 3-13, I-50019, Sesto Fiorentino, Italy

Index

Chemicals *S2*

Evaluation of CA-inhibitory activity *S2*

Crystallization and X-ray data collection *S3*

Summary of Data Collection and Atomic Model Refinement Statistics for hCA II *S4*

Figure S1 *S5*

In silico studies *S6*

References *S7*

Chemicals

AT-121 (purity ≥95%; Item No. 26150) was supplied by Cayman-Chemical, Michigan, USA. Acetazolamide (purity ≥99%) weas supplied by Sigma–Aldrich, Milan, Italy. All other reagents were of analytical grade.

Evaluation of CA-inhibitory activity

An Applied Photophysics stopped-flow instrument was used to assay the CA-catalysed CO_2-hydration activity[1]. Phenol red (at a concentration of 0.2 mM) was used as an indicator, working at an absorbance maximum of 557 nm, with 20 mM HEPES pH 7.5 as buffer and 20 mM sodium sulfate (to maintain a constant ionic strength), following the initial rates of the CA-catalyzed CO_2-hydration reaction for a period of 10–100 s. The CO_2 concentrations ranged from 1.7 to 17 mM for determination of the kinetic parameters and inhibition constants[2]. For each inhibitor, at least six traces of the initial 5–10% of the reaction were used to determine the initial velocity. The uncatalyzed rates were determined in the same manner and were subtracted from the total observed rates. Stock solutions of inhibitor (0.1 mM) were prepared in distilled deionized water and dilutions of up to 0.01 nM were made in the assay buffer. Inhibitor and enzyme solutions were pre-incubated together for 15 min at room temperature prior to the assay in order to allow formation of the enzyme–inhibitor complex. The inhibition constants were obtained by nonlinear least-squares methods using Prism 3 and the Cheng–Prusoff equation, as reported previously, and represent the mean from at least three different determinations. All CA isoforms were recombinant isoforms obtained in-house, as reported previously[3-5].

Crystallization and X-ray data collection

Crystal of hCA II was obtained using the hanging drop vapor diffusion method using 24 well Linbro plate. 2 µl of 10 mg/ml solution of hCA II in Tris-HCl 20 mM pH 8.0 were mixed with 2 µl of a solution of 1.5 M sodium citrate, 0.1 M Tris pH 8.0 and were equilibrated against the same solution at 296 K. The complex was prepared by soaking the native crystal in the mother liquor solution containing the inhibitor at concentration of 10 mM for one day. The crystal was flash-frozen at 100K using a solution obtained by adding 15% (v/v) glycerol to the mother liquor solution as cryoprotectant. Data on crystal of the complex was collected using synchrotron radiation at the ID30A-1 beamline at European Synchrotron Radiation Facility ESRF (Grenoble, France) with a wavelength of 0.965459Å and a PILATUS4 4M detector. Data were integrated and scaled using the program XDS[6]. Data processing statistics are shown in supporting information.

Structure determination

The crystal structure of hCA II (PDB accession code: 4FIK) without solvent molecules and other heteroatoms was used to obtain initial phases using Refmac5[7]. 5% of the unique reflections were selected randomly and excluded from the refinement data set for the purpose of Rfree calculations. The initial |Fo - Fc| difference electron density maps unambiguously showed the inhibitor molecules. The inhibitor was introduced in the model with 0.5 occupancy for each conformation. Refinements proceeded using normal protocols of positional, isotropic atomic displacement parameters alternating with manual building of the models using COOT[8]. The quality of the final models was assessed with COOT and RAMPAGE[9]. Crystal parameters and refinement data are summarized in Electronic Supplementary Information (ESI). Atomic coordinate was deposited in the Protein Data Bank (PDB accession code: 29IR). Graphical representations were generated with Chimera[10].

Table S1. Summary of Data Collection and Atomic Model Refinement Statistics for hCAII

	**hCAII + AT-121**
PDB ID	29IR
Wavelength (Å)	0.965459
Space Group	P21
Unit cell (a, b, c, α, β, γ)(Å,°)	42.32, 41.46, 72.0, 90.00, 104.38, 90.00
Limiting resolution (Å)	69.82-1.27 (1.29-1.27)
Unique reflections	61104 (2600)
Rmerge (%)	5.2 (73.3)
Rmeas (%)	7.3 (103.1)
Redundancy	2.3 (2.3)
Completeness overall(%)	94.9 (77.9)
<I/σ(I)>	8.4 (1.1)
CC (1/2)	0.992 (0.328)
**Refinement statistics**	
Resolution range(Å)	69.82-1.27
Rfactor (%)	14.43
Rfree(%)	17.84
r.m.s.d. bonds(Å)	0.0098
r.m.s.d. angles (°)	1.8361
**Ramachandran statistics** (%)	
Most favored	97.3
additionally allowed	2.7
outlier regions	0.0
**Average B factor ($Å^2$)**	
All atoms	19.228
Inhibitors	30.591
Solvent	30.713

Figure S1:

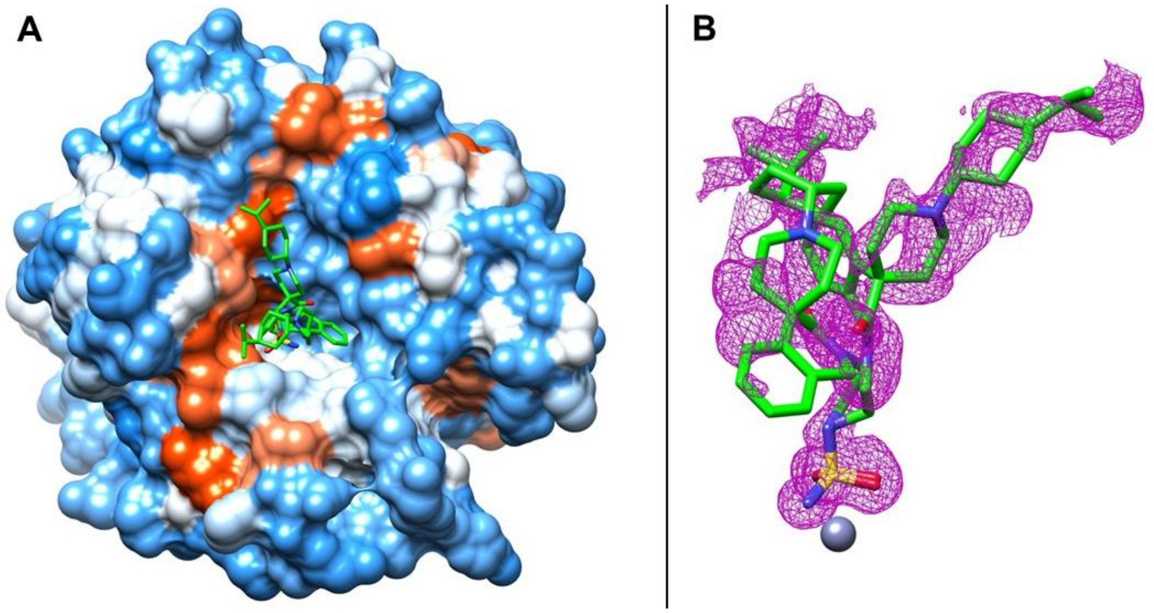

Figure S1. **A)** Complex of AT-121 with hCA II (PDB: 29IR). The surface of hCAs are visualized through the "hydrophobicity surface" preset, from blue for the most hydrophilic, to white, to red for the most hydrophobic **B)** Electron density of inhibitor AT-121 bound to zinc (grey) in hCA II active site. $2F_o$-F_c maps and contoured to the 1.0 σ level.

***In silico* studies**

Crystal structures of hCA I (PDB: 1AZM)[11], hCA III (PDB: 3UYQ)[12], hCA IV (PDB: 5JN8)[13], hCA VII (PDB: 3ML5)[14], hCA IX (PDB: 5FL4)[15], hCA XII (PDB: 1JD0)[16], hCA XIII (PDB: 3CZV)[17], and hCA XIV (PDB: 4LU3)[18] were retrieved by Protein Data Bank[19], while the structures of hCA VA, VB and VI were downloaded by Alphafold[20]. All 3D-structures used for computational studies were prepared using the Protein Preparation Wizard tool implemented in the Schrödinger[21] suite, assigning bond orders, adding hydrogens, deleting water molecules, and optimizing H-bonding networks. The energy minimization protocol with a Root Mean Square Deviation (RMSD) value of 0.30 Å was applied using an Optimized Potentials for Liquid Simulation (OPLS4) force field[22]. The 3D ligand structures were prepared by Maestro[21a] and evaluated for their ionization states at pH 7.4 ± 0.5 with Epik[21b]. The conjugate gradient method in Macromodel was used for energy minimization (maximum iteration number: 2500; convergence criterion: 0.05 Kcal/mol/Å2)[21c]. Grids for docking were centered on the centroid of complex ligands. For molecular docking studies, the software Glide SP (default settings) was used. The standard precision (SP) mode of the Glide Score function was applied to evaluate the predicted binding poses of all series[21d]. The best docking poses for each compound were scored for its binding free energies (dG bind) with the Prime MM-GBSA protocol, module implemented in Maestro Schrödinger suite,[21e] using a VSGB solvation model and enabling residue flexibility 3Å around the ligand[23]. Figures were generated with Chimera[10].

References

1. Khalifah, R.G. The carbon dioxide hydration activity of carbonic anhydrase. I. Stop flow kinetic studies on the native human isoenzymes B and C. *J. Biol. Chem*. **1971**, 246, 2561-2573.
2. Supuran CT. Carbonic anhydrases: novel therapeutic applications for inhibitors and activators. *Nat Rev Drug Discov.* **2008**, 7, 168-181.
3. Tanini D, Capperucci A, Ferraroni M, Carta F, Angeli A, Supuran CT. Direct and straightforward access to substituted alkyl selenols as novel carbonic anhydrase inhibitors. *Eur J Med Chem*. **2020**, 185, 111811.
4. Angeli A, Vaiano F, Mari F, Bertol E, Supuran CT. Psychoactive substances belonging to the amphetamine class potently activate brain carbonic anhydrase isoforms VA, VB, VII, and XII. *J Enzyme Inhib Med Chem*. **2017**, 32, 1253-1259.
5. Angeli A, Tanini D, Capperucci A, Malevolti G, Turco F, Ferraroni M, Supuran CT. Synthesis of different thio-scaffolds bearing sulfonamide with subnanomolar carbonic anhydrase II and IX inhibitory properties and X-ray investigations for their inhibitory mechanism. *Bioorg Chem.* **2018**, 81, 642-648.
6. Leslie, A.G.W., Powell, H.R. Procesing diffraction data with mosflm. In: Read RJ, Sussman JL (eds) Evolving methods for macromolecular crystallography, vol 245, NATO Science series, Springer, Dordrecht, **2007**, pp. 41-51.
7. Murshudov, G.N., Vagin, A.A., Dodson, E.J. Refinement of macromolecular structures by the maximum-likelihood method. *Acta Crystallogr D Biol Crystallogr*. **1997**, 53, 240-255.
8. Emsley, P., Lohkamp, B., Scott, W., Cowtan, K. Features and development of Coot. *Acta Crystallogr D Biol Crystallogr.* **2010**, 66, 486-501.
9. Lovell, S.C., Davis, I.W., Arendall III, W.B., de Bakker, P.I.W., Word, J.M., Prisant, M.G., Richardson, J.S., Richardson, D.C., Structure validation by Cα geometry: ϕ,ψ and Cβ deviation. *Proteins*, **2003**, 50, 437-450.
10. Pettersen, E.F., Goddard, T.D., Huang, C.C., Couch, G.S., Greenblatt, D.M., Meng, E.C., Ferrin, T.E., UCSF Chimera—a visualization system for exploratory research and analysis, *J. Comput. Chem.*, **2004**, 25, 1605-1612.
11. Chakravarty S, Kannan KK. Drug-protein interactions. Refined structures of three sulfonamide drug complexes of human carbonic anhydrase I enzyme. *J Mol Biol.* **1994**;243:298-309.

12. 12. Elder I, Fisher Z, Laipis PJ, Tu C, McKenna R, Silverman DN. Structural and kinetic analysis of proton shuttle residues in the active site of human carbonic anhydrase III. *Proteins.* **2007**;68:337-43.
13. Mickevičiūtė A, Timm DD, Gedgaudas M, Linkuvienė V, Chen Z, Waheed A, Michailovienė V, Zubrienė A, Smirnov A, Čapkauskaitė E, Baranauskienė L, Jachno J, Revuckienė J, Manakova E, Gražulis S, Matulienė J, Di Cera E, Sly WS, Matulis D. Intrinsic thermodynamics of high affinity inhibitor binding to recombinant human carbonic anhydrase IV. *Eur Biophys J.* **2018**;47:271-290.
14. Di Fiore A, Truppo E, Supuran CT, Alterio V, Dathan N, Bootorabi F, Parkkila S, Monti SM, De Simone G. Crystal structure of the C183S/C217S mutant of human CA VII in complex with acetazolamide. *Bioorg Med Chem Lett*. **2010**;20:5023-6.
15. Leitans J, Kazaks A, Balode A, Ivanova J, Zalubovskis R, Supuran CT, Tars K. Efficient Expression and Crystallization System of Cancer-Associated Carbonic Anhydrase Isoform IX. *J Med Chem*. **2015**;58:9004-9.
16. Whittington DA, Waheed A, Ulmasov B, Shah GN, Grubb JH, Sly WS, Christianson DW. Crystal structure of the dimeric extracellular domain of human carbonic anhydrase XII, a bitopic membrane protein overexpressed in certain cancer tumor cells. *Proc Natl Acad Sci U S A*. **2001**;98:9545-50.
17. Di Fiore A, Monti SM, Hilvo M, Parkkila S, Romano V, Scaloni A, Pedone C, Scozzafava A, Supuran CT, De Simone G. Crystal structure of human carbonic anhydrase XIII and its complex with the inhibitor acetazolamide. *Proteins*. **2009**;74:164-75.
18. Alterio V, Pan P, Parkkila S, Buonanno M, Supuran CT, Monti SM, De Simone G. The structural comparison between membrane-associated human carbonic anhydrases provides insights into drug design of selective inhibitors. *Biopolymers*. **2014**;101:769-778.
19. H.M. Berman, J. Westbrook, Z. Feng, G. Gilliland, T.N. Bhat, H. Weissig, I.N. Shindyalov, P.E. Bourne, The Protein Data Bank. *Nucleic Acids Research*. **2000**;28:235-242.
20. Jumper J, Evans R, Pritzel A, Green T, Figurnov M, Ronneberger O, Tunyasuvunakool K, Bates R, Žídek A, Potapenko A, Bridgland A, Meyer C, Kohl SAA, Ballard AJ, Cowie A, Romera-Paredes B, Nikolov S, Jain R, Adler J, Back T, Petersen S, Reiman D, Clancy E, Zielinski M, Steinegger M, Pacholska M, Berghammer T, Bodenstein S, Silver D, Vinyals O, Senior AW, Kavukcuoglu K, Kohli P, Hassabis D. Highly accurate protein structure prediction with AlphaFold. *Nature*. **2021**;596:583-589.

21. Schrödinger Suite Release 2024-3, Schrödinger, LLC, New York, NY, 2024: (a) Maestro v.14.1; (b) Epik, v.6.9; (c) Macromodel v.14.5; (d) Glide, v.10.4; (e) Prime, v.5.5; (f) Impact, v.10.4; (g) Jaguar, v.12.5; (h) Desmond, v.7.9; (i) QikProp v.4.6.
22. Lu C, Wu C, Ghoreishi D, Chen W, Wang L, Damm W, Ross GA, Dahlgren MK, Russell E, Von Bargen CD, Abel R, Friesner RA, Harder ED. OPLS4: Improving Force Field Accuracy on Challenging Regimes of Chemical Space. J Chem Theory Comput. 2021;17:4291-4300.
23. Kalinin S, Nocentini A, Kovalenko A, Sharoyko V, Bonardi A, Angeli A, Gratteri P, Tennikova TB, Supuran CT, Krasavin M. From random to rational: A discovery approach to selective subnanomolar inhibitors of human carbonic anhydrase IV based on the Castagnoli-Cushman multicomponent reaction. *Eur J Med Chem*. **2019**, 182:111642.
